# Virologists are “Symbionts” in Microbial Ecology

**DOI:** 10.1264/jsme2.ME3104rh

**Published:** 2016-12

**Authors:** Ken Takai

**Affiliations:** 1 Department of Subsurface Geobiological Analysis and Research (D-SUGAR), Japan Agency for Marine-Earth Science and Technology (JAMSTEC)2–15 Natsushima-cho, Yokosuka, Kanagawa 237–0061Japan

Virologists are now getting arms for exploration of a wide spectrum of virosphere in this planet. The arms mean techniques through the next generation sequence for virome (28, 34) and may be compared as revolutionary appearance of ribosomal RNA (rRNA) gene sequencing for microbial ecologists in 1990’s (14, 17, 19). This research highlight introduces trends in environmental virology and current topics achieved by virologists who are establishing a kind of “symbiotic” relationship with microbial ecologists.

The research history of viruses is relatively ‘new’ when compared to that of prokaryotes. It is well known, however, that bacteriophage studies have greatly contributed to the development of modern molecular biology. Virus research began in the 1890’s with discovery of an acute infectious agent in tobacco causing leaf spots (2). Since then, many viruses have been found as the causal agents for animal and plant diseases and microbial lysis and other phenomena. Not limited to plague-associated viruses, viruses that do not show any of negative interactions with their hosts (*e.g.*, disease and lysis) have been also found from plants (4, 31), insects (22), and fungi (18, 27). As for both Gram-positive and -negative bacteria, filamentous phages including M13 do not cause host killing or lysis but they are assembled at and secreted across the cell membrane (33). In 1990’s, pioneer studies showed that virus-like particles occur in great abundance (ca. 10^7^ particles per milliliter of seawater) (16). These findings have clarified that there are a great number of viruses or virus-like particles in environments and have pointed that the viruses so far explored by virologists are only the tip of the iceberg of the Earth’s virosphere.

With overwhelming development of nucleic acid sequencing technology in this decade, virologists have been able to explore viruses in natural environments without isolation and microscopic observation (15). With newly developed technologies, the genetic diversity of viruses in environments, particularly free-existing viral particles independent of their hosts, has been extensively investigated. In the course of genetic exploration of viral diversity, double-stranded (ds) DNA viruses have been identified from many of the viromes such as in sea water (7), marine sediment (5) and human (6) and equine (8) faces. Obviously, other types of viruses including single-stranded (ss) DNA viruses and ssRNA viruses have also been identified from viromes such as in seawater (1, 11, 12), subseafloor sediment (39), insects (32), and plants (30). Several excellent reviews cover those latest findings (28, 34). Nevertheless, the metagenomic approach to the environmental viruses has substantial drawbacks: technical difficulty in addressing their functions and in exploration of viral elements lying behind their hosts (13, 30, 32).

Virologists in Japan have provided significant impacts on the investigations of functional environmental viruses and the intracellular viral elements. The DNA viruses that would be involved in retreat of red tide algaes (24, 25) and the RNA virus infecting dinoflagellates (26, 35, 38) represent excellent examples of functional environmental viruses in aquatic ecosystems. In addition, although it is not yet justified as true viruses, there are pioneering reports on intracellular genetic elements that are of great interest as a gene transfer agent (9, 10). Recently, technical developments for simultaneous fractionation of four kinds of viral nucleic acids (ssDNA, dsDNA, ssRNA, dsRNA) (36) and exhaustive identification of non-retro RNA viruses (37) have been reported. These two methods would be powerful tools for metagenomic approach to yet unseen viromes in the near future. The newly developed method by Urayama *et al.* (37), named FLDS, stands for fragmented and loop primer ligated dsRNA sequencing, overcomes one of the substantial technical difficulties in viromic studies: mining viruses lying behind their hosts without any of negative interactions. FLDS is based on the fact that long dsRNA molecules are known to be RNA virus-specific molecules and molecular markers for RNA virus infection and replication (3, 23). The method can enrich non-retro RNA viruses from any of the biological samples and comprehensively identify the full-length genomes of RNA viruses (37). Indeed, a total of 22 full-length genomes of the novel RNA viruses have been identified from only 1 g (wet weight) of diatom community (37). This method is already used for characterizing nonpathogenic RNA virus in insects (20), and will rapidly renew the RNA virus list in various organisms in the future. Even in the rapidly developed areas such as marine and gut viromes, FLDS will catch not only the extracellular but also the intracellular viruses that may be significantly associated with the microbial communities and even the host animals.

Viruses have long been recognized as the agent to kill their hosts, but some are known to have the function to confer profitable properties on their hosts (21, 29), comparable to the bacteria, which have both pathogenic and symbiotic traits in their hosts. Viruses are now being regarded as one of the genetic elements that may alter the properties of hosts and endow the community with new functions. A research initiative called as “Neo-virology” in Scientific Research on Innovative Areas of Grant-in-Aid for Scientific Research (Kakenhi) (http://neo-virology.org/) is now ongoing in Japan. With a current perspective of environmental microbiology, microbial ecologists are being “infected” with virologists forming a strong symbiotic partnership.
